# Nutrient patterns and cardiometabolic risk factors among Iranian adults: Tehran lipid and glucose study

**DOI:** 10.1186/s12889-020-08767-6

**Published:** 2020-05-11

**Authors:** Mohammad Mottaghian, Pantea Salehi, Farshad Teymoori, Parvin Mirmiran, Firoozeh Hosseini-Esfahani, Fereidoun Azizi

**Affiliations:** 1 Student Research committee, Department of Clinical Nutrition and Dietetics, Faculty of Nutrition and Food Technology, Shahid Beheshti University of Medical Sciences, Tehran, Iran; 2 Department of Clinical Nutrition and Dietetics, Faculty of Nutrition and Food Technology, Shahid Beheshti University of Medical Sciences, Tehran, Iran; 3 Nutrition and Endocrine Research Center, Research Institute for Endocrine Sciences, Shahid Beheshti University of Medical Sciences, P.O. Box: 1985717413, Tehran, Iran; 4 Department of Nutrition, School of Public Health, Iran University of Medical Sciences, Tehran, Iran; 5 Endocrine Research Center, Research Institute for Endocrine Sciences, Shahid Beheshti University of Medical Sciences, Tehran, Iran

**Keywords:** Nutrient pattern, Principal component analysis, Fasting blood sugar (FBS), Triglycerides (TGs), High density lipoprotein-cholesterol (HDL), Low density lipoprotein-cholesterol (LDL), Total cholesterol (TC), Blood pressure (BP)

## Abstract

**Background:**

The present study aimed to assess the relation between nutrient patterns and changes in adult anthropometric and cardiometabolic factors.

**Methods:**

This study was conducted on 1637 adults participating in the Tehran Lipid and Glucose Study (2005–2008), who were free of cardiovascular diseases and cancer and had completed dietary data. They were followed to the next survey (2008–2011). Dietary intakes were collected and nutrient patterns were obtained. Three year changes in anthropometric and cardiometabolic factors were measured.

**Results:**

Five nutrient patterns were extracted. The first pattern was characterized by “plant protein, thiamine, niacin, and minerals including phosphorus, zinc, copper, magnesium, manganese, and selenium”. Animal protein, lactose, vitamin D, riboflavine, pantothenic acid, vitamin B12, calcium, phosphorus, and zinc" were loaded in the second pattern. The third and fourth patterns were characterized by “vitamin K, fiber, calcium, iron, manganese, and potassium”, and “high correlation with starch, thiamine and folate, and negative correlation with mono and poly unsaturated fatty acids and vitamin E”, respectively. The fifth pattern was high in Fructose, vitamins A, C, pyridoxine, and potassium. There was no association between nutrient patterns and 3-year changes in blood pressure and fasting blood glucose; whereas, per each quartile increment of the fifth pattern adjusted for potential confounders, triglyceride change was decreased [β = − 3.66, 95% CI (− 6.57, − 0.57); P for trend = 0.014].

**Conclusion:**

Present study indicates that nutrient patterns may have an association with cardiometabolic factors, particularly a pattern rich in fructose, vitamins A, C, pyridoxine, and potassium which decreases triglyceride level.

## Background

Cardiovascular diseases (CVD) are known as one of the most important causes of mortality and obstacles for sustainable development of human societies in the world [1]. In 2015, the estimated global rate of CVD and its mortality were 422.7 and 17.9 million, respectively [2]. Various risk factors of CVD such as hypertension, dyslipidemia, and hyperglycemia have been recognized previously [3]. Recent studies warn about worldwide trends in increasing blood pressure (BP), body mass index (BMI), serum cholesterol, and blood sugar, particularly in low and middle income countries [4–7].

Dietary factors are one of the main determinants of CVD risk factors. Diet-disease relationships were investigated in the form of dietary patterns from 1980, in this form the pooled effect of whole foods in the diet on different diseases is analyzed [8, 9]. Dietary patterns present a wide view and rational insight of the relation between diet and disease, and can predict the risk of various diseases.

Although dietary patterns based on foods have more applicability in communities, but there are different kinds of foods with various cooking and preservation methods in the world; also, dietary patterns are unable to determine the mechanisms through which main nutrients are related to whole body homeostasis [10]. However, nutrients are dietary components which perform the effects of diet, their composition is the same in the entire world, and not affected by different cooking methods and food preservation [10]. Several recent studies used alternative methods of dietary patterns which pooled effects of various nutrients instead of foods and investigated the association of main dietary nutrient patterns with the risk of chronic disease [11–16]. In this approach dietary intake of nutrients are calculated using food composition databases and then the group of nutrients which have high correlation with each other determines as dietary nutrient patterns using statistical methods [14].

Salehi-Abargouei et al. extracted 3 nutrient patterns related to central obesity in adults; the second pattern (thiamine, niacin, betaine, folate, iron, selenium, and starch) and third pattern (glucose, fructose, sucrose, fiber, C and K vitamins, and copper) were associated with lower and higher risk of central obesity, respectively [14]. In another study, two nutrient patters including animal derived nutrients, and also, starch and folate were associated with higher body mass index [12]. Other studies extracted different nutrient patterns related to some chronic diseases and biochemical factors such as hemoglobin A1c (HbA1c) and fasting blood sugar [15–17]. However, cohort studies investigating the association of nutrient patterns and prospective changes in cardiometabolic factors are scarce. The current prospective study aimed to investigate the association of nutrient patterns with cardio metabolic risk factors including lipid profiles, BP, and blood sugar in adult participants.

## Methods

### Subjects

Subjects of this cohort study were selected among Tehran lipid and glucose study (TLGS) participants, an ongoing population-based prospective study with 3-year interval measurements which began in 1991 in Tehran, the capital of Iran [18].

Participants who aged 30–75 years entered in the third survey (2005–2008), and had complete dietary data were entered the study and followed up for 3-years to the fourth survey (2011–2014). Subjects who had chronic disease, special diet or consumed anti-hypertensive, anti-hyperglycaemic, and lipid lowering drugs, over- and under-reporters of energy intake, pregnant and lactating women, and who had missing data on measurements at baseline or follow up were excluded from the study participants..

### Dietary intake assessment

Dietary intakes were assessed using a valid and reliable semi-quantitative food frequency questionnaire (FFQ) which previously validated for the TLGS study. The reliability and validity of the FFQ has been previously reported [19]. During face-to-face interview by trained and experienced dieticians, the consumption frequency of each food item during the previous year was collected on a daily, weekly, or monthly basis. Portion sizes of consumed foods, reported in household measures were then converted into grams. The dietary intakes of energy and nutrients were computed using the United States Department of Agriculture (USDA) food composition table (FCT), and the Iranian FCT only for local food items that were not available in USDA FCT.

### Physical activity assessment

The modified physical activity questionnaire which previously validated for participants of the TLGS study was used for assessing physical activity levels. The validity and reliability of this was reported earlier [20]; Interviewers asked participants to report the frequency and time spent on activities of light, moderate, hard, and very hard intensity during the past 12 months, according to a list of common activities of daily life. Physical activity levels were expressed as metabolic equivalent hours per week.

### Clinical and biological measurements

Trained interviewers collected information on age, sex, medical history, medication use, and smoking habits using pretested questionnaires. The participant’s weight, height, waist circumference (WC), were measured based on standard protocols as previously described [21]. Body mass index (BMI) was calculated as weight (Kg) divided by height squared (m^2^).

After a 12–14-h overnight fasting, between 7:00 and 9:00 a.m., blood samples were taken while subjects were in a sitting position, then transferred into vacutainer tubes. Blood samples were centrifuged within 30 to 45 min of collection. Details of the biochemical analyses and measurements including triglycerides (TGs), Serum total cholesterol (TC), high-density lipoprotein-cholesterol (HDL-C), and low density lipoprotein cholesterol (LDL-C), fasting blood sugar (FBS), and blood pressure (BP) have been reported previously [18, 20, 21].

### Statistical analysis

Data analyses were conducted using the Statistical Package for Social Sciences (version 20.0; SPSS Inc., Chicago IL). The normality of variables was assessed using histogram charts and Kolmogorov–Smirnov analysis. Baseline characteristics of subjects were expressed as mean ± SD or median (25–75 interquartile range) for continuous variables, and percentages for categorical variables.

**Fig. 1:**
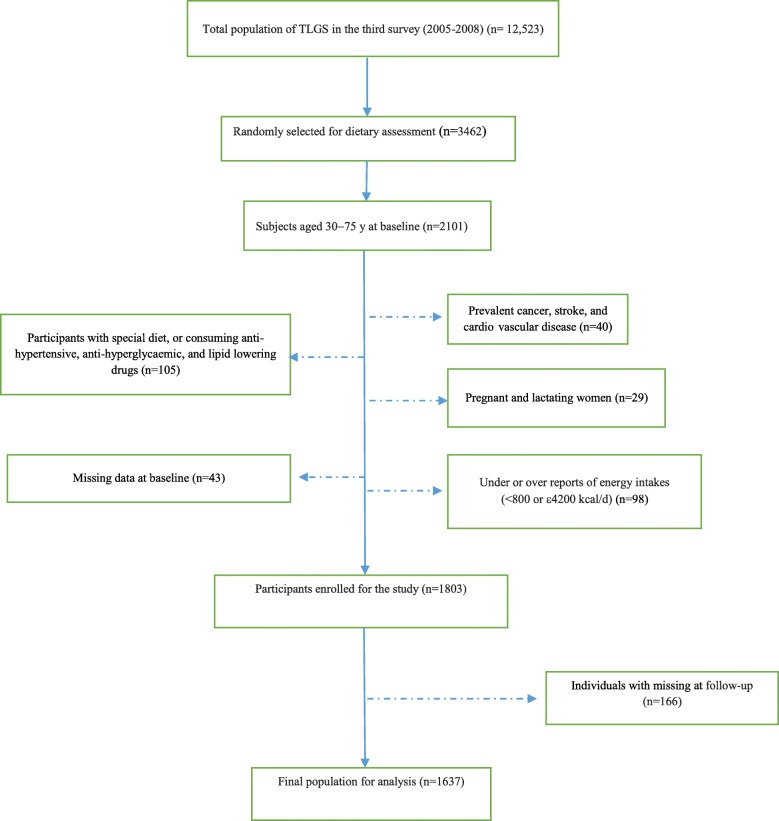
The diagram of the study participants and follow-up

The 36 main nutrient intakes of participants at baseline of study were chosen based on their consumption values and literature reviews on their potential association with cardiometabolic factors. Nutrient patterns were derived using principal component analysis (PCA) with varimax rotation and correlation matrix at baseline. Statistical correlation between variables and adequacy of sample size was tested, using the Bartlett test of sphericity (*P* < 0.001) and the Kaiser-Mayer-Olkin test (0.70). Factor scores of each extracted factors were calculated by summing the frequency of consumption multiplied by factor loadings across 36 nutrient items. We identified five dominant factors based on scree plot (Fig. 1) (eigenvalue > 2) and categorized factor scores into quartiles. The 3 year changes of FBS, SBP, DBP, serum TG, HDL-C, LDL-C, and TC were computed by subtracting follow-up from their baseline values. Multiple linear regression analysis was conducted with FBS, BP, and lipid profile changes as dependent variables and quartiles of nutrient patterns as independent variables.

Analysis was conducted using three models of adjustment for potential confounders. The first model was adjusted for age and sex. The second was adjusted for model 1 and BMI, physical activity, and smoking. And the last model includes model 2 and daily energy intake, education levels, marital and employment status. Furthermore, for each of dependent variables, the corresponding value in the baseline survey was adjusted. Data were analysed using SPSS statistics software version 20. Beta coefficients (unstandardized) and their respective confidence intervals 95% (95% CI) were reported, and *P*-values < 0.05 were considered as statistically significant.

## Results

Of 12,523 participants who entered in the third survey (2005–2008), 3462 were randomly selected and agreed to complete the dietary assessment, of these, 2101 participants aged 30–75 years were included and followed up to the fourth survey (2011–2014) (Fig. 1). After applying the exclusion criteria, 1803 participants enrolled for the study at baseline; they were followed for 3 years and 166 subjects had missing data on the follow-up assessments. Finally, 1637 participants remained for the final analysis (follow up rate, 90.7%).

The mean age and BMI of participants (45.8% male) were 46.6 years and 28.0 Kg/m2, respectively. The median (IQR, 25, 75) of 3 years changes of SBP and DBP were 3.0 (− 5.0, 11.0) and 3.0 (− 2.0, 10.0) mm Hg, respectively. Furthermore, the median (IQR, 25, 75) change values for FBS, TG, HDL, LDL, and TC were 7.0 (1.0, 13.0), − 3.0 (− 36.0, 26.0), 5.0 (1.0, 9.0), − 3.2 (− 17.8, 11.8), 2.0 (− 16.0, 18.0) mg/dl, respectively.

**Fig. 2:**
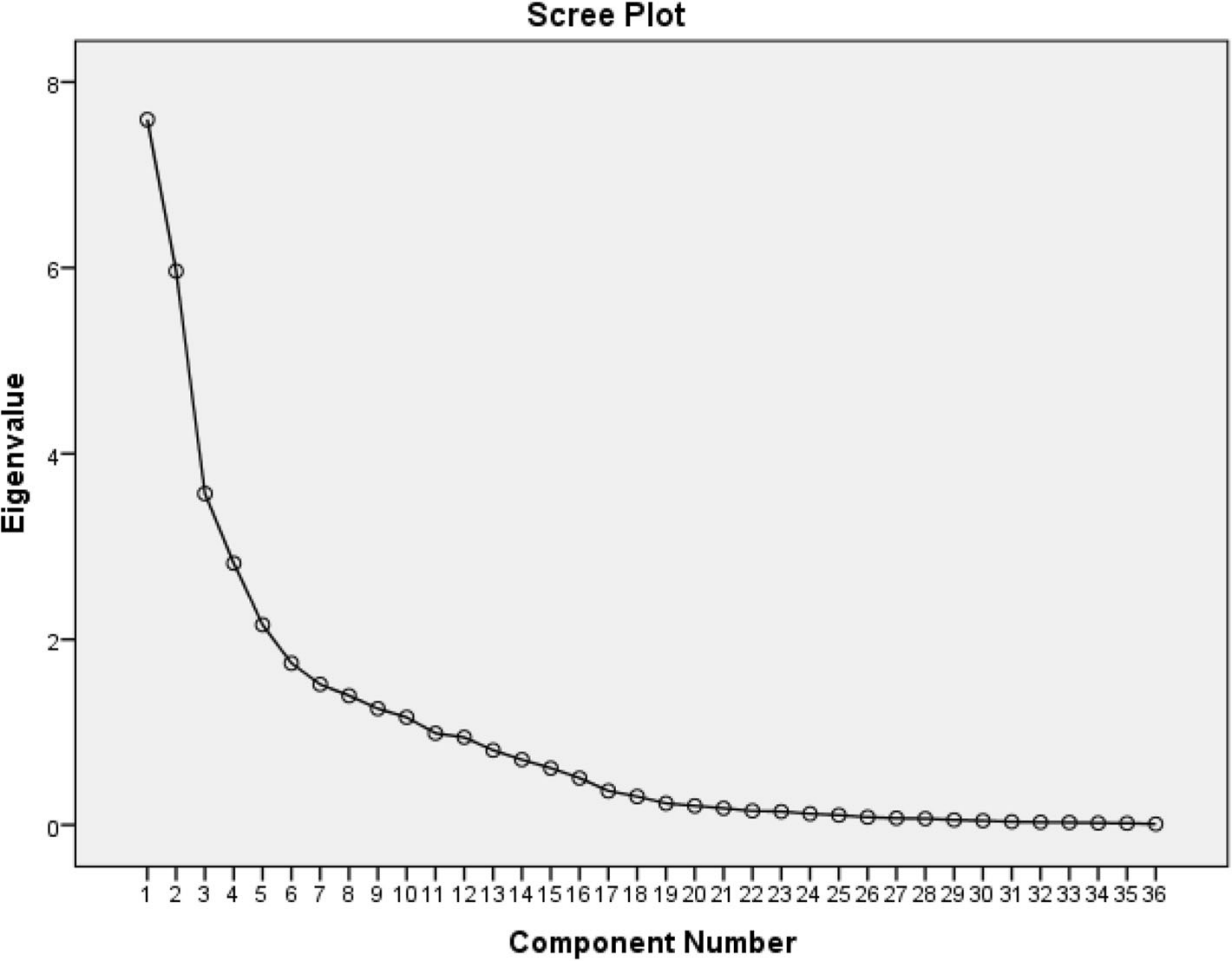
Scree plot for extraction of dietary nutrient patterns by principal component analysis. The 36 dietary nutrients was used as input variables and nutrient patterns based on eigenvalues > 2 were identified as main nutrient patterns

Table 1 shows factor loading matrix of 36 nutrients and explains variances of each of five nutrient patterns. Using factor analysis, five dominant patterns (Fig. 2) were identified which explained 62/2% of total variations of 36 main nutrient intakes. The first pattern, characterized by plant proteins, thiamine, niacin and minerals including phosphorus, zinc, copper, magnesium, manganese and selenium had 22% coverage of total variance. The nutrients including animal protein, lactose, vitamin D, riboflavin, pantothenic acid, vitamin B12, calcium, phosphorus and zinc were highly loaded in the second pattern. The third pattern had the highest loading for vitamin K, fiber, calcium, iron, manganese and potassium. The fourth pattern had positive correlation with starch, thiamine and folate, and high negative correlation with mono and poly-unsaturated fatty acids and vitamin E. The fifth pattern was characterized by fructose, vitamins A and C, pyridoxine and potassium.

**Table 1:** Factor loading matrix and explained variances for major nutrient patterns identified by factor analysis in 1637 participants aged 30–75 years of Tehran Lipid and Glucose Study (2006–2008) ^a b^

Nutrient patterns					
	Pattern 1	Pattern 2	Pattern 3	Pattern 4	Pattern 5
**Nutrients**					
Starch				**0.40**	
Sucrose					
Lactose		**0.90**			
Fructose					**0.55**
Glucose					
Animal protein		**0.52**			
Plant protein	**0.80**				
Fiber					
Saturated fatty acids			**0.40**		
Mono unsaturated fatty acids				**−0.84**	
Poly unsaturated fatty acids				**−0.83**	
cholesterol					
Vitamin A					**0.64**
Vitamin D		**0.73**			
Vitamin E				**−0.78**	
Vitamin K			**0.94**		
Thiamine	**0.55**			**0.53**	
Riboflavine		**0.75**			
Niacin	**0.52**	**−0.32**			0.38
Pantothenic acid		**0.64**	0.32		**0.46**
Pyridoxine			0.36	**0.58**	
Folate					
Vitamin B12		**0.41**			
Vitamin C			**0.64**		**0.86**
Calcium		**0.71**			
Phosphor	**0.42**	**0.72**			
Iron			**0.93**		
Zinc	**0.60**	**0.42**	0.32		
Copper	**0.66**				
Magnesium	**0.75**				
Manganese	**0.66**		**0.51**		
Chromium					
Selenium	**0.88**				
Sodium					
Potassium		0.38	**0.55**		**0.67**
Caffeine					
**Explained variance (%)**	22.0	17.2	9.5	6.8	6.4
**Cumulative explained variance (%)**	22.0	39.3	48.8	55.7	**62.2**

^a^Principle Component Analysis (PCA) performed on 36 nutrients adjusted for total energy intake

Nutrients with loadings > 0.40 and less than −0.40 (in bold) are being characteristic for the five patterns; loadings less than 0.3 (in absolute value) are suppressed

^b^Kaiser’s Measure of Sampling Adequacy, KMO = 0.70, Bartlett’s test of sphericity = < 0.001

General characteristics of participants based on quartiles of nutrient patterns are shown in Table 2. Across quartiles of the first pattern, the mean age, male percentage, BMI and WC, FBS, SBP, DBP, and TGs increased (*P* < 0.05). Also, for the second pattern, HDL-C increased across quartiles 1 to 4; while, male percentage and smokers reduced (*P* < 0.001). With increasing quartiles of the third pattern, the mean of FBS increased and male percentage decreased (*P* < 0.05). Across quartiles of the fourth pattern, the percentage of men, mean age and SBP of participants increased and BMI decreased (*P* < 0.05). The mean age of participants, BMI, SBP and HDL-C across quartiles of the fifth pattern increased (*P* < 0.05); however, the percentage of smokers, men, married individuals, high education levels, and the mean of TGs decreased (*P* < 0.05).

**Table 2:** Baseline characteristics of 1637 participants 30–75 year old of the Tehran Lipid and Glucose Study (2006–2008) across quartiles of nutrient patterns *

	Pattern 1		Pattern 2		Pattern 3		Pattern 4		Pattern 5	
	Q 1(*n* = 407)	Q 4(*n* = 408)	Q 1(*n* = 408)	Q 4(*n* = 407)	Q 1(*n* = 408)	Q 4(*n* = 407)	Q 1(*n* = 408)	Q 4(*n* = 407)	Q 1(*n* = 408)	Q 4(*n* = 407)
Age (years)	45.5 ± 10.6	48.7 ± 11.1^‡^	46.4 ± 10.4	47.0 ± 11.2	45.7 ± 11.3	47.0 ± 11.1	45.2 ± 9.9	47.5 ± 11.6^‡^	45.0 ± 10.4	48.4 ± 11.5^†^
Men (%)	40.5	53.0^†^	53.3	40.6^†^	52.8	43.3^‡^	34.7	61.7^†^	54.6	38.9^†^
Body mass index (kg.m2)	27.7 ± 4.4	28.4 ± 4.8^‡^	31.1 ± 5.1	28.0 ± 4.3	27.9 ± 4.5	28.0 ± 4.7	28.2 ± 4.6	27.6 ± 4.2^‡^	27.4 ± 4.3	28.4 ± 4.6^‡^
Waist circumference (Cm)	91.5 ± 11.5	94.8 ± 11.7^†^	93.9 ± 11.9	92.7 ± 11.4	93.0 ± 12.1	92.8 ± 11.9	92.7 ± 12.3	93.7 ± 10.4	92.7 ± 11.6	92.0 ± 12.3
Smoking (%)	14.9	11.5	17.1	10.6^†^	12.2	12.9	11.3	17.4	16.6	9.1^†^
Physical activity (MET.h.week)	27.7(12.1–53.5)	24.3(10.3–55.5)	27.5(10.0–58.0)	27.5(11.0–55.5)	26.2(10.2–57.3)	26.5(11.9–53.9)	26.7(11.9–54.0)	27.4(10.4–56.3)	27.7(10.5–57.0)	26.5(12.0–55.5)
Marital status (% of married)	64.5	59.4	60.8	63.0	63.9	62.8	64.3	59.8	63.4	57.6^‡^
Education levels (higher than diploma)	28.0	31.4	26.1	30.2	33.4	29.9	31.0	29.6	31.8	27.0^‡^
Employment status (% of employed)	72.0	67.5	70.7	71.7	72.9	72.4	72.9	68.3	73.5	65.1^†^
**Blood pressure**										
Systolic blood pressure (mmHg)	113.2 ± 17.5	116.6 ± 17.6^‡^	115.5 ± 16.9	114.8 ± 17.8	113.9 ± 15.7	115.0 ± 17.2	112.6 ± 17.4	116.7 ± 17.1^†^	114.6 ± 17.4	116.2 ± 18.5^†^
Diastolic blood pressure (mmHg)	74.5 ± 9.9	76.0 ± 11.1^‡^	75.9 ± 11.5	74.8 ± 9.9	75.0 ± 10.5	75.2 ± 10.6	73.9 ± 11.1	76.6 ± 10.0	75.5 ± 10.4	75.3 ± 10.6
**Biochemical factors**										
Diastolic blood pressure (mmHg)	74.5 ± 9.9	76.0 ± 11.1^‡^	75.9 ± 11.5	74.8 ± 9.9	75.0 ± 10.5	75.2 ± 10.6	73.9 ± 11.1	76.6 ± 10.0	75.5 ± 10.4	75.3 ± 10.6
Triglycerides (mg.dl)	147.0 ± 86.2	164.3 ± 93.4^‡^	159.2 ± 93.1	154.3 ± 89.1	151.6 ± 87.0	162.0 ± 93.6	151.4 ± 82.3	168.0 ± 94.9	168.9 ± 109.4	152.5 ± 78.5^‡^
High density lipoprotein cholesterol (mg.dl)	41.9 ± 10.0	40.9 ± 9.6	40.7 ± 9.8	42.6 ± 10.5^‡^	41.3 ± 9.8	42.1 ± 10.1	42.1 ± 9.6	40.4 ± 10.1	40.9 ± 10.0	42.3 ± 10.2^‡^
Low density lipoprotein cholesterol (mg.dl)	123.4 ± 32.9	120.4 ± 33.5	121.6 ± 34.8	122.1 ± 31.6	121.9 ± 34.2	120.8 ± 30.3	122.4 ± 29.5	123.4 ± 35.5	125.1 ± 31.4	121.7 ± 36.6
Total cholesterol (mg.dl)	194.2 ± 37.9	194.0 ± 39.1	193.5 ± 40.1	194.7 ± 36.6	192.9 ± 39.0	194.3 ± 35.5	194.5 ± 34.4	195.7 ± 39.7	197.7 ± 38.0	194.4 ± 40.1

^*^Data represented as mean ± SD or median (interquartile 25–75) and percent

P for trend: ^†^ < 0.001; ^‡^ < 0.05. P trend was calculated using general linear models for continuous variables or Chi-square tests for categorical variables

Table 3 showed dietary intake of macronutrients and food groups across quartiles of nutrient patterns. With increasing quartiles of the first pattern, carbohydrate, protein, fiber, grain, vegetable, white meat, and legume intakes increased (*P* < 0.001); whereas, fat, fruit, dairy and simple sugar consumptions decreased (*P* < 0.001). Across quartiles of the second pattern energy intake, protein, fat, fruit, vegetable, dairy, red meat and legume consumption increased (*P* < 0.05); however, the intake of carbohydrate, fiber, grain and simple sugar decreased (*P* < 0.05). Dietary protein, fiber, vegetable, dairy, white meat, and legume increased across quartiles of the third pattern (*P* < 0.05); whereas, grain and simple sugar intakes decreased (*P* < 0.05). With increasing quartiles of the fourth pattern, intake of carbohydrate, protein, fiber, grain, fruit, and dairy products increased (*P* < 0.05); however, fat, vegetable, red meat, and legume decreased (*P* < 0.05). Dietary intake of carbohydrate, protein, fiber, fruit, vegetable, and legume increased across quartiles of the fifth pattern (*P* < 0.05); whereas, intake of energy, fat, grain, red meat and simple sugar decreased (*P* < 0.001).

**Table 3:** Dietary intakes in 1637 participants 30–75 year old of Tehran Lipid and Glucose Study (2006–2008) across quartiles of nutrient patterns *

	Pattern 1		Pattern 2		Pattern 3		Pattern 4		Pattern 5	
	Q 1(*n* = 407)	Q 4(*n* = 408)	Q 1(*n* = 408)	Q 4(*n* = 407)	Q 1(*n* = 408)	Q 4(*n* = 407)	Q 1(*n* = 408)	Q 4(*n* = 407)	Q 1(*n* = 408)	Q 4(*n* = 407)
Energy (kcal)	2526 ± 654	2337 ± 702	2107 ± 715	2398 ± 651^†^	2456 ± 728	2366 ± 674	2378 ± 719	2351 ± 715	2543 ± 709	2311 ± 703^‡^
Carbohydrates (% of energy)	55.6 ± 7.9	60.3 ± 6.5^†^	59.7 ± 8.0	56.3 ± 6.6^†^	59.7 ± 7.4	58.3 ± 6.9	52.2 ± 6.5	63.5 ± 5.9^†^	56.3 ± 7.9	60.9 ± 6.4^†^
Total protein (% of energy)	12.2 ± 1.9	15.0 ± 2.2^†^	12.6 ± 2.5	14.8 ± 2.0^†^	13.2 ± 2.0	14.4 ± 2.4^†^	12.8 ± 2.3	13.9 ± 2.3^†^	13.4 ± 2.3	13.9 ± 2.5^‡^
Total fat (% of energy)	34.4 ± 7.6	27.9 ± 5.6^†^	30.0 ± 8.0	31.5 ± 6.0^‡^	29.7 ± 7.2	30.4 ± 6.5	37.8 ± 6.3	24.6 ± 4.6^†^	31.7 ± 7.8	29.1 ± 6.1^†^
Fiber (g/1000 kcal)	14.9 ± 6.3	19.0 ± 6.5^†^	17.5 ± 7.4	15.9 ± 6.0^‡^	14.6 ± 4.8	20.6 ± 7.9^†^	15.1 ± 5.7	19.0 ± 9.2^†^	17.1 ± 9.4	18.7 ± 5.6^†^
Grains (g/d)	420 ± 195	527 ± 246^†^	486 ± 260	412 ± 199^†^	512 ± 274	477 ± 208^‡^	394 ± 204	575 ± 284^†^	601 ± 278	374 ± 177^†^
Fruits (g/d)	435 ± 350	343 ± 257^†^	336 ± 284	382 ± 281^‡^	440 ± 338	375 ± 284	346 ± 270	395 ± 315^‡^	177 ± 112	646 ± 351^†^
Vegetables (g/d)	308 ± 187	345 ± 241^†^	269 ± 174	352 ± 228^†^	287 ± 199	389 ± 212^†^	327 ± 213	299 ± 244^‡^	214 ± 116	448 ± 256^†^
Dairy (g/d)	553 ± 316	427 ± 265^†^	201 ± 124	769 ± 299^†^	456 ± 303	501 ± 305^†^	431 ± 256	483 ± 326^‡^	507 ± 333	471 ± 290
Red and processed meat (g/d)	26.5(15.2–41.9)	24.5(13.6–42.5)	21.6(13.3–41.3)	27.9(13.4–47.2) ^‡^	25.7(14.1–41.3)	25.2(14.9–41.5)	25.5(15.2–42.5)	22.3(12.2–39.4) ^‡^	28.5(14.1–47.6)	24.0(13.2–38.4) ^†^
White meats (g/d)	42.5(27.4–65.1)	47.9(31.9–72.1) ^†^	42.1(26.1–67.3)	45.6(31.5–65.9)	44.9(30.2–68.1)	47.9(32.8–70.4) ^‡^	45.4(27.9–66.3)	42.8 (28.9–67.1)	43.3(27.4–65.9)	45.6(31.6–68.0)
Legume and nuts (g/d)	16.4(9.9–27.7)	20.1(10.4–37.3) ^†^	15.5(8.0–29.3)	17.4(10.4–31.4) ^‡^	16.7(9.5–30.8)	20.0(11.0–36.7) ^‡^	18.8(11.2–34.1)	14.5(8.9–26.2) ^‡^	14.7(8.5–28.0)	20.2(11.8–34.7) ^†^
Sweets and simple sugars	82.2(52.6–145.0)	48.4(27.0–78.2) ^†^	62.4(38.1–103.2)	51.6(29.3–84.5) ^‡^	67.4(37.7–122.0)	56.3(31.5–90.2) ^†^	63.2(35.4–109.3)	59.5(32.0–99.3)	68.5(39.7–113.0)	52.0(26.1–86.7) ^†^

^*^Data represented as mean ± SD or median (interquartile 25–75) and percent

P for trend: ^†^ < 0.001; ^‡^ < 0.05. P trend was calculated using general linear models

The association of nutrient patterns and 3 year changes in FBS, SBP, and DBP are shown in Table 4. There were no associations between quartiles of nutrient patterns with changes in FBS, SBP, and DBP in crude and adjusted models.

**Table 4:** Multiple linear regression analysis evaluating the association between each quartile increase of nutrient patterns and changes in fasting blood sugar, systolic and diastolic blood pressure after three years of follow up in 1637 participants of the Tehran Lipid and Glucose Study

	Fasting blood sugar changes		Systolic blood pressure changes		Diastolic blood pressure changes	
	*β*^a^*(95% CI)*	*P* value	*β (95% CI)*	*P* value	*β (95% CI)*	*P* value
**Pattern 1**						
Model1^b^	0.894 (− 0.056, 1.843)	0.065	− 0.210 (− 0.750, 0.323)	0.444	− 0.149 (− 0.532, 0.234)	0.446
Model2^c^	0.791 (− 0.159, 1.742)	0.103	− 0.336 (− 0.900, 0.167)	0.178	− 0.264 (− 0.643, 0.115)	0.172
Model3^d^	0.659 (− 0.298, 1.615)	0.177	− 0.320 (− 0.817, 0.176)	0.206	− 0.205 (− 0.550, 0.140)	0.243
**Pattern 2**						
Model1^b^	− 0.221 (−1.163, 0.720)	0.645	− 0.205 (− 0.743, 0.334)	0.456	−0.110 (− 0.492, 0.273)	0.574
Model2^c^	−0.217 (− 1.157, 0.723)	0.651	− 0.195 (− 0.726, 0.337)	0.472	−0.114 (− 0.492, 0.263)	0.552
Model3^d^	−0.019 (− 0.973, 0.937)	0.968	−0.210 (− 0.710, 0.291)	0.411	−0.188 (− 0.536, 0.160)	0.289
**Pattern 3**						
Model1^b^	0.146 (−0.804, 1.095)	0.763	0.113 (−0.429, 0.655)	0.683	0.056 (−0.328, 0.441)	0.774
Model2^c^	0.152 (−0.795, 1.099)	0.753	0.100 (−0.434, 0.634)	0.713	0.059 (−0.328, 0.431)	0.789
Model3^d^	0.140 (−0.806, 1.086)	0.772	0.122 (−0.372, 0.615)	0.629	0.068 (−0.275, 0.412)	0.695
**Pattern 4**						
Model1^b^	0.064 (−0.889, 1.017)	0.895	−0.174 (− 0.719, 0.372)	0.533	− 0.150 (− 0.538, 0.238)	0.448
Model2^c^	0.123 (− 0.828, 1.074)	0.800	− 0.113 (− 0.651, 0.425)	0.680	− 0.097 (− 0.479, 0.286)	0.620
Model3^d^	0.047 (− 0.904, 0.999)	0.922	−0.147 (− 0.644, 0.351)	0.563	− 0.068 (− 0.414, 0.279)	0.702
**Pattern 5**						
Model1^b^	−0.026 (− 0.988, 0.935)	0.957	0.085 (− 0.465, 0.635)	0.762	0.036 (− 0.354, 0.426)	0.857
Model2^c^	−0.108(− 1.069, 0.853)	0.825	− 0.002 (− 0.545, 0.540)	0.993	−0.032 (− 0.417, 0.354)	0.871
Model3^d^	−0.166 (− 1.128, 0.796)	0.735	− 0.089 (− 0.592, 0.413)	0.727	− 0.099 (− 0.448, 0.250)	0.579

^a^ Beta regression coefficient; the positive β values indicated that higher adherence to nutrient patterns increases the changes in dependent variables and vice versa

^b^Adjusted for age and sex

^c^Adjusted for model 1 and body mass index, physical activity, and smoking (yes or no)

^d^ Adjusted for model 2 and energy intake, education levels (under diploma, diploma and associate degree, bachelor and higher), marital status (single, married), and employment status (employed, unemployed). For blood pressure and fasting blood sugar changes, their corresponding values in baseline phase were adjusted

Table 5 shows the association between nutrient patterns and 3-year changes in lipid profiles. In age and sex adjusted model, per each quartile increment of the fifth nutrient pattern, the beta (β) coefficient (95% CI) of TGs change was − 3.40 (− 6.30, − 0.49); P for trend = 0.022. In the final adjusted model, the beta (β) (95% CI) of TG change was − 3.66 (− 6.57, − 0.57); P for trend = 0.014. The present study showed no statistically significant relation between other nutrient patterns and the serum lipid profiles.

**Table 5:** Multiple linear regression analysis evaluating the association between each quartile increase of nutrient patterns and changes in lipid profile after three years of follow up in 1637 participants of the Tehran Lipid and Glucose Study

	Triglyceride changes		HDL-cholesterol changes		LDL-cholesterol changes		Total cholesterol	
	*β*^a^*(95% CI)*	*P* value	*β*^a^*(95% CI)*	*P* value	*β*^a^*(95% CI)*	*P* value	*β*^a^*(95% CI)*	*P* value
**Pattern 1**								
Model1^b^	− 1.16 (− 4.02, 1.69)	0.425	− 0.11 (− 0.43, − 0.19)	0.471	− 0.45 (− 1.59, 0.68)	0.436	− 0.98 (− 2.25, 0.29)	0.131
Model2^c^	− 1.24 (− 4.11, 1.62)	0.395	− 0.11 (− 0.42, − 0.20)	0.491	− 0.30 (− 1.45, 0.83)	0.601	− 0.85 (− 2.13, 0.42)	0.192
Model3^d^	− 1.28 (− 4.17, 1.65)	0.382	− 0.11 (− 0.43, − 0.20)	0.476	− 0.28 (− 1.43, 0.86)	0.627	− 0.86 (− 2.15, 0.42)	0.191
**Pattern 2**								
Model1^b^	1.37 (− 1.47, 4.22)	0.343	0.02 (− 0.28, 0.33)	0.871	0.71 (−0.42, 1.84)	0.218	0.84 (−0.42, 2.11)	0.191
Model2^c^	1.46 (−1.38, 4.30)	0.314	0.02 (−0.29, 0.33)	0.898	0.76 (−0.37, 1.89)	0.187	0.88 (−0.38, 2.15)	0.172
Model3^d^	1.77 (−1.12, 4.66)	0.231	0.07 (−0.24, 0.39)	0.661	0.62 (−0.52, 1.78)	0.286	0.85 (−0.44, 2.15)	0.195
**Pattern 3**								
Model1^b^	−0.29 (−3.16, 2.57)	0.839	−0.12 (− 0.43, 0.19)	0.454	0.27 (− 0.86, 1.41)	0.637	−0.03 (−1.31, 1.24)	0.957
Model2^c^	−0.32 (− 0.319, 2.54)	0.825	− 0.11 (− 0.43, 0.19)	0.471	0.26 (− 0.87, 1.40)	0.647	−0.05 (− 1.33, 1.22)	0.937
Model3^d^	−0.36 (− 3.23, 2.50)	0.802	− 0.12 (− 0.44, 0.18)	0.430	0.29 (− 0.84, 1.44)	0.607	−0.03 (− 1.31, 1.24)	0.957
**Pattern 4**								
Model1^b^	0.03 (− 2.85, 2.91)	0.983	−0.08 (− 0.40, 0.23)	0.604	0.60 (− 0.54, 1.75)	0.303	0.86 (− 0.42, 2.14)	0.189
Model2^c^	0.12 (−2.75, 3.00)	0.931	−0.08 (− 0.40, 0.23)	0.605	0.58 (− 0.56, 1.73)	0.316	0.87 (− 0.40, 2.16)	0.181
Model3^d^	0.02 (−2.86, 2.91)	0.987	−0.10 (− 0.42, 0.21)	0.525	0.63 (− 0.51, 1.79)	0.278	0.89 (− 0.39, 2.18)	0.175
**Pattern 5**								
Model1^b^	−3.40 (−6.30, −0.49)	0.022	0.16 (−0.15, 0.48)	0.313	−0.71 (−1.86, 0.44)	0.227	−0.96 (−2.25, 0.34)	0.148
Model2^c^	−3.51 (−6.24, − 0.60)	0.018	0.16 (− 0.15, 0.48)	0.303	− 0.61 (− 1.78, 0.54)	0.299	−0.88 (− 2.18, 0.42)	0.185
Model3^d^	−3.66 (− 6.57, − 0.74)	0.014	0.14 (− 0.18, 0.46)	0.392	− 0.56 (− 1.73, 0.60)	0.341	−0.88 (− 2.18, 0.42)	0.186

^a^Beta regression coefficient; the positive β values indicated that higher adherence to nutrient patterns increases the changes in dependent variables and vice versa

^b^Adjusted for age and sex

^c^Adjusted for model 1 and body mass index, physical activity, and smoking (yes or no)

^d^Adjusted for model 2 and energy intake, education levels (under diploma, diploma and associate degree, bachelor and higher), marital status (single, married), and employment status (employed, unemployed). For each of lipid profile factors, its corresponding value in baseline phase was adjusted

## Discussion

In this 3-year prospective study among participants of the TLGS, we identified five nutrient patterns using principal component analysis. We observed no association between nutrient patterns with prospective changes in FBS, SBP, DBP, HDL, LDL, and TC. However, the risk of TG change decreased in participants who had higher adherence to the fifth pattern, which was characterized by fructose, vitamins A and C, pyridoxine and potassium.

### Nutrient patterns and prospective changes in BP and FBS

To our knowledge, this study was the first to investigate the association of nutrient patterns with prospective changes in FBS and BP, and observed no significant association. However, the relation between nutrient patterns extracted from factor analysis and serum glucose and BP levels investigated in some cross-sectional studies [15, 16]. Chikowore et al. conducted a gender base analysis on 2010 urban and rural black participants from South Africa, and identified three nutrient patterns [16]. The pattern characterized by complex carbohydrates, fiber, and B group vitamins, and a pattern which was rich in thiamin, zinc and plant proteins were associated with lower serum glucose and HbA1c, in rural women and men, respectively. Differences in study populations, races, and study design are considerable factors which could justify inconsistent findings of these two studies.

At baseline of our study, participants who had higher adherence to the first, fourth, and fifth nutrient patterns had higher unadjusted values of SBP levels. Also across quartiles of the first and third patterns, the FBS values increased. However, in our study patterns did not make significant changes in FBS and BP after 3-years of follow up after adjusting for potential confounders; It is valuable to mention that a nutrient factor predicts a lower amount of cardiometabolic factor changes compared with main factors such as age, sex, and body mass index; also nutrient patterns have many interactions which attenuates or reinforces the overall effect of the pattern. If more nutrients in a pattern have similar direction in applying their physiological effects, the overall effect of the pattern is strengthening, and vice versa. Furthermore, mechanisms which regulate serum glucose and BP are highly sensitive, and maintain their levels in a normal range [22], in comparison to weight which is easily influenced by dietary energy and fat intakes. Although the association of single nutrient such as sodium, calcium, potassium, magnesium [23], and amino acids [21, 24] with BP, and simple sugars, fructose, etc. [25] with FBS has been proved previously; but when the association of whole nutrients with FBS and BP were evaluated, the interaction of useful, inactive and potentially harmful nutrients is inevitable and their effects may be neutralized by each other. Also in the general population most people have normal FBS and BP, while they consume all nutrients from their diet.

### Nutrient pattern and prospective changes of lipid profiles

In the current study, the fifth pattern, which was characterized by nutrients rich in fruit and vegetables, was inversely related to 3-year changes in TGs, while other patterns showed no significant relationship with serum levels of lipid profiles. At baseline, individuals became older and more obese across quartiles of the fifth pattern; however, they had lower levels of TGs. The positive role of the fifth pattern components including vitamin A, vitamin C, and high intake of fruit and vegetable in reducing the risk of metabolic syndrome was shown in Korean adults [26]. In Australian middle aged men, a nutrient pattern rich of vitamin A and antioxidant agents existing in fruit, had a significant reverse relation with CRP and inflammation [27]. The association of nutrient patterns and lipid profiles in the form of metabolic syndrome has been previously investigated [15]. Khayyatzadeh et al. indicated that patterns with high simple carbohydrates such as fructose, increased the level of TGs in women; however, patterns with high selenium, vitamin A and B groups decreased TG levels in men [15]. Fructose had a high factor loading in the fifth pattern of our study. The direct association of dietary fructose, consumed from high-fructose corn syrup and sweetened beverages, with TG levels observed [28, 29]. Review articles on clinical trials had shown that fructose in an isocaloric diet does not increase TG levels compared to other carbohydrates; however, if the calorie intake increases, fructose increases the synthesis of TG. Generally, there is no definitive finding that fructose necessarily increases TG levels compared to other carbohydrates [30, 31]. In our fifth pattern, fructose intake was mostly supplied by fruits, not beverages. The conservative effect of fruit and vegetable consumption on TGs levels has been proven. The other nutrient patterns had no significant relationship with changes in serum lipid profiles which may be due to interactions between nutrient components For example, in the third pattern, fiber can reduce the absorption of divalent metals including calcium, iron, and manganese [32]; these nutrients seem to attenuate the effects of each other. Whereas, nutrients of the fifth pattern, such as vitamin c, fructose and pantothenic acid, have boosting effects on some biological actions in the human body [33, 34].

What matters is founding nutrient patterns which significantly have beneficial association with cardiometabolic factors in epidemiological studies and then testing these patterns in different communities and consequently focusing on a nutrient pattern as complementary treatment for prevention of cardiometabolic complications.

The present study has valuable strengths, including the prospective design, relatively high sample size, and high accuracy in nutritional data measurement which was collected by trained and experienced dieticians compared with previous studies that mostly used self-reported questionnaires. There are also limitations to this study; despite the adjustment of many possible confounders, we do not rule out the possibility of unknown confounders affecting the finding of this study; also, since identification of patterns in this study was done using factor analysis, the limitations of this type of analysis can also be our research constraints. The number of factors derived from the factor analysis are largely influenced by the decisions of the researchers through the number of nutrients entering the analysis, which effect the factor loading of nutrients in each pattern, the method for obtaining the data matrix (rotation method or other methods) and selecting the number of factors (patterns) [9]. However, we selected a wide range of main nutrients which may have effects on cardiometabolic factors; also, five predominant patterns had explained 62.2% of the total variations in dietary intakes of main nutrients.

## Conclusions

In summary, the findings of the present study showed that consuming a pattern of nutrients rich in vitamin A, vitamin C, pyridoxine, potassium and fructose or a pattern rich of nutrients originates from fruit and vegetable is associated with a reduction in serum TG.

## Data Availability

The datasets used and/or analysed during the current study are available from the corresponding author on reasonable request.

## Abbreviations

TLGS: Tehran Lipid and Glucose Study
CVD: Cardiovascular diseases
BMI: Body mass index
BP: Blood pressure
FFQ: Food frequency questionnaire
USDA: United States Department of Agriculture
FCT: Food composition Table
WC: Waist circumference
TG: Triglyceride
HDL-C: High-density lipoprotein-cholesterol
LDL-C: Low-density lipoprotein-cholesterol
TC: Total cholesterol
FBS: Fasting blood sugar
PCA: Principal component analysis
95% CI: 95% Confidence interval
